# Allergy to Thaumatin-like Proteins—What Do We Know?

**DOI:** 10.3390/foods14040543

**Published:** 2025-02-07

**Authors:** Magdalena Rydzyńska, Zbigniew Bartuzi, Tomasz Rosada, Magdalena Grześk-Kaczyńska, Natalia Ukleja-Sokołowska

**Affiliations:** 1 Department and Clinic of Allergology, Clinical Immunology and Internal Diseases, Jan Biziel University Hospital No. 2 in Bydgoszcz, 85-168 Bydgoszcz, Poland; magdalenagrzesk@gmail.com; 2 Department and Clinic of Allergology, Clinical Immunology and Internal Diseases, Ludwik Rydygier Collegium Medicum in Bydgoszcz, Nicolaus Copernicus University in Toruń, 87-100 Toruń, Poland; zbartuzi@cm.umk.pl (Z.B.); medtom@op.pl (T.R.); ukleja@10g.pl (N.U.-S.)

**Keywords:** allergy, food allergy, allergen, thaumatin-like protein

## Abstract

Thaumatin-like proteins (TLPs) are a class of allergens that are predominantly found in fruits. These proteins are involved in plant defense mechanisms and exhibit antifungal activity. TLPs are classified as pathogenesis-related proteins (PR-5) and are typically synthesized in response to biotic stress, such as pathogen attacks. Sensitization to TLPs can result in a broad spectrum of allergic reactions, ranging from localized symptoms, such as oral allergy syndrome, to severe manifestations, including anaphylaxis. Key allergens within this group include Mal d 2 (apple), Pru p 2 (peach), and Pru av 2 (cherry). The list of allergens belonging to the TLP protein group continues to expand with newly discovered molecules. Diagnostic approaches for TLP allergies remain limited. Allergen component-resolved diagnostics can detect specific TLPs. The epidemiology of TLP-induced allergies is underexplored, and further research is needed to elucidate the prevalence, natural course, and clinical outcomes of these allergic conditions.

## 1. Introduction

Fruits are a common cause of food allergies. The most important allergens in fruits are lipid transfer proteins, profilins, PR-10 family proteins, and gibberellin-regulated proteins. Less well-known allergens include thaumatin-like proteins (TLPs). Sensitization to TLPs can cause a wide range of symptoms, including anaphylaxis [1,2,3]. TLPs represent a poorly understood group of proteins, and the list of described allergens is limited. The existing knowledge primarily relies on case reports, with a notable lack of systematic studies addressing this issue. Additionally, the number of available diagnostic tools is limited, resulting in inadequate recognition of this allergy, despite its potentially life-threatening course.

Furthermore, there is a lack of statistical data assessing the prevalence of this allergy. This is a particularly significant issue that remains insufficiently addressed in the literature. A search of the PubMed database using the term “allergy to thaumatin-like proteins” yielded 70 results, of which 32 articles were published in the last decade, none of which were meta-analyses or systematic reviews. A search for “thaumatin-like protein sensitization” returned 17 results. From these, 31 articles were selected for detailed analysis.

In light of these limitations, further research is essential to gain a more comprehensive understanding of this allergy, its pathophysiological mechanisms, and its health implications. Accurate classification and diagnosis will be critical not only for advancing medical knowledge but also for improving the quality of life of patients who are affected by this condition.

The objective of this article is to review the current state of knowledge regarding TLPs. To the best of our knowledge, this is the first review article on the role of thaumatin-like proteins as allergens.

## 2. Thaumatin-like Proteins

Thaumatin is a sweet-tasting protein that was first isolated from the fruit of the African plant *Thaumatococcus daniellii* over 50 years ago [4,5]. Thaumatin-like proteins were named due to the homology of their amino acid sequence to that of this peptide. Their molecular weight ranges from 10 to 34 kDa. They are polypeptides composed of approximately 200 amino acid residues, and their structure contains 16 cysteine residues, which stabilize the molecule through eight disulfide bridges. As a result, they are resistant to high temperatures and changes in pH. They belong to the group of pathogenesis-related proteins, PR5. These proteins are divided into three groups: those that are secreted in response to infection, osmotic changes, and those that are involved in antifungal defense. Their synthesis is stimulated under conditions of plant stress and pathogen attack [5]. They exhibit antifungal properties by binding to β-1,3-glycans, components of the fungal cell wall [6]. Diagrams illustrating the structure of selected TLPs can be found in the cited research works [7,8].

## 3. Allergy to TLPs

The mechanism underlying IgE-dependent hypersensitivity reactions is known by the rapid onset of symptoms following allergen exposure. In the initial phase, antigen-presenting cells (APCs), such as dendritic cells, recognize and process foreign molecules, subsequently presenting them to Th2 helper lymphocytes. Activated Th2 lymphocytes secrete cytokines, including interleukins IL-4 and IL-13, which stimulate the activation of B lymphocytes. In response, B lymphocytes differentiate into plasma cells and produce specific IgE antibodies (sIgE). These IgE antibodies bind to FcεRI receptors on the surface of mast cells and basophils, rendering them sensitized. Upon re-exposure to the same allergen, the allergen binds to IgE that is anchored on the surface of these cells, leading to their activation. This process triggers degranulation, determined by the release of granule contents such as histamine, tryptase, prostaglandins, and leukotrienes. These mediators elicit typical allergic manifestations, including bronchospasm, pruritus, mucosal swelling, and erythema [9,10].

Previously documented cases of allergy to TLPs exhibited symptoms such as oral allergy syndrome, rhinoconjunctivitis, bronchospasm, diarrhea, and anaphylaxis following the consumption of fruits. These findings highlight the role of IgE-dependent mechanisms in the immune response to proteins belonging to the TLP family. In many instances, the identification and isolation of TLPs were possible due to their ability to bind sIgE that is present in the serum of allergic patients. To date, no evidence has been reported supporting the existence of alternative mechanisms for inducing hypersensitivity reactions that are independent of IgE.

Allergies can occur through both the oral and inhalation routes. TLPs may cause cross-reactions between pollen and fruit [11].

## 4. TLP Sources

The list of allergens belonging to the TLP protein group continues to expand with newly discovered molecules. Below is a brief characterization of the allergens from this protein family that have been described so far. A summary of the fundamental information regarding TLPs discovered to date is provided in Table 1.

### 4.1. Food Allergens

#### 4.1.1. Mal d 2

This is the first described food allergen belonging to the TLP group. This protein, with a molecular weight of 23 kDa, is found in the fruit of the domestic apple tree; both the flesh and skin of the fruit contain it [13,14]. It is resistant to digestion and thermal processing [15]. It has antifungal properties. Recombinant Mal d 2 showed antifungal activity against *Fusarium oxysporum* and *Penicillium expansum* [16]. In a study of 34 apple-allergic patients conducted by Hsieh et al., 75% of the patients had specific IgE directed against Mal d 2 [13]. In another study, which included 389 apple-allergic patients from Spain, Italy, the Netherlands, and Austria, sensitization to Mal d 2 was observed in 15% of Italian and Spanish residents and 5% of those living in the Netherlands and Austria [17,18].

#### 4.1.2. Pru p 2

Another representative of the TLP protein family is the peach Pru p 2 allergen, whose molecular weight ranges from 25 to 28 kDa. Three isoforms of Pru p 2 have been identified. A group of 31 patients with a history of allergic reactions after peach ingestion, who had positive prick skin tests with peach extract and a positive oral provocation test with this fruit, were analyzed. ELISA immunoenzymatic tests and basophil activation tests were performed. Pru p 2.0201 was positive in 80% of the BAT results and in 77% of the ELISA results. Pru p 2.0101 was responsible for positive BAT results in 50% of the patients and 71% of the ELISA results. Pru p 2.0301 was responsive in 50% of the subjects. Pru p 2 was also reported to bind to anti-CCD antibodies [19]. Palacín et al. evaluated 16 proteins derived from the TLP family, assessing their cross-reactivity. Sera from 329 patients, all from Spain, were examined using a microarray method. For 10 allergens, the presence of antibodies was detected in less than 10% of the patients. Antibodies against Pru p 2.0201 were the most common. Peach, chestnut, and plane pollen TLPs were used in the inhibition test. Pru p 2 inhibited the binding of IgE antibodies to chestnut and plane pollen [11].

#### 4.1.3. Pru av 2

The Pru av 2 allergen of *Prunus avium* (cherry), which is a TLP, has a molecular weight of 23 kDa and is the main allergen of cherries [20]. An increasing number of reports have indicated the involvement of the epidermal barrier in the development of food allergies. The penetration of allergens through the skin induces the synthesis of specific IgE. This pathway for the acquisition of hypersensitivity was demonstrated in mice that were sensitized to the Pru av 2 cherry allergen. In the study, cherry extract was applied to the skin on the backs of the mice. Changes in the concentration of cherry-specific IgG1 and IgE were then evaluated by ELISA. Using the immunoblotting method, cherry antigens causing transdermal sensitization were identified. A 27 kDa protein was detected, which is a thaumatin-like protein—the Pru av 2 panallergen cherry antigen. Based on the collected results, it was concluded that the thaumatin-like protein, Pru av 2, is the cherry allergen that is responsible for transdermal sensitization. Extracts of this fruit, when present in cosmetics, can cause food allergies via a porous epidermal barrier [21].

#### 4.1.4. Cap a 1

*Capsicum annuum*, an annual bell pepper, is the source of an allergen that is a TLP. The protein, Cap a 1, has a molecular weight of 23 kDa. It was isolated by analyzing the serum of 22 patients with mugwort–birch–celery syndrome [22].

#### 4.1.5. Act d 2

The 24 kDa allergen of the kiwi fruit, *Actinidia chinensis*, is another representative of TLPs. The analysis of seven cases of patients with oral allergy symptoms after kiwi consumption contributed to its isolation. It occurs in two isoforms. The TLP of kiwi fruit has been shown to be glycosylated and to have antifungal activity against *Saccharomyces cerevisiae* and *Candida albicans* [23]. It has been demonstrated that the content of the allergenic protein and the ratio of its isoforms in the fruit depend on its maturity and developmental stage [24]. It cross-reacts with fruit proteins from the same family, such as Mal d 2 and Pru av 2 [11]. In addition, Act d 2 has been shown to share epitopes with Fes p 4 from *Festuca pratensis*, resulting in cross-reactivity [25]. There is a case report that describes exercise-induced, meal-dependent anaphylaxis preceded by Act d 2 sensitization [1]. In a study evaluating 10 children with a confirmed allergy to kiwi (by oral challenge), 50% of the patients showed reactivity to Act d 2 [26]. The role of N-glycosylation in allergic reactivity and cross-reactivity was also examined. Act d 2 was deglycosylated, and its effect on dendritic cells was assessed. It has been proven that the occurrence of an allergic reaction depends on the peptide component [7].

#### 4.1.6. Mus a 4

The thaumatin-like protein of banana, Mus a 4, has a molecular weight of 20 kDa. A group of 51 pediatric patients with clinical symptoms after banana ingestion, positive skin prick tests, and the presence of banana-specific IgE were evaluated. It was estimated that up to 72% of them had antibodies against Mus a 4 [27].

### 4.2. Inhalant Allergens

#### 4.2.1. Ole e 13

The TLP of the fruit of the *European olive* has a molecular weight of 23 kDa, and sensitization occurs via the inhalation route. There have been cases where olive TLP was the cause of occupational asthma in oil mill workers. The fruit extract that is produced during the process was responsible for the symptoms [28]. Most interestingly, patients with asthma did not experience discomfort after consuming olives. Fruits that are consumed undergo a maceration process. It has been shown that macerated fruits do not contain TLPs. TLPs have also not been detected in olive pollen [29].

#### 4.2.2. Jun a 3

Jun a 3 is an inhalant allergen with a mass of 30 kDa. In a study conducted by U.S. researchers, sera from patients who were allergic to mountain cedar or Japanese cedar were evaluated for IgE binding to Jun a 3. Of the 14 patients who were sensitive to mountain cedar, 6 reacted to Jun a 3, and of the 36 patients who were sensitive to Japanese cedar, 12 reacted to Jun a 3. Cross-reactivity between mountain cedar and Japanese cedar was demonstrated. In addition, it has been shown that the Jun a 3 content in mountain cedar pollen varies from year to year in the same plants and from region to region [30].

#### 4.2.3. Cry j 3

Japanese cedar is a source of a TLP with a molecular weight of 27 kDa. It is a species of cedar that is one of the most common causes of pollinosis among the Japanese population. One hundred patients who were diagnosed with Japanese cedar pollen allergy, based on clinical symptoms and the presence of specific IgE, were studied. It was shown that antibodies directed against Cry j 3 were present in 27 patients [31]. In addition, testing the sera of five patients with oral allergy symptoms, using RAST inhibition and immunoblot methods, showed cross-reactivity between Japanese cedar pollen and tomato fruit [32]. Cry j 3 shows high homology with the Jun a 3 allergen, sharing 86% of its sequence identity. Furthermore, the sequence homology of TLP Cry j 3 is 45% identical with Mal d 2, 44% with Pru av 2, 50% with Act d 2, and 47% with Cap a 1 [10].

#### 4.2.4. Cup s 3

TLP of common cypress has a molecular mass of 34 kDa and is an inhalant allergen. Its isolation was made possible by analyzing a group of 23 patients with confirmed inhalant allergies to the pollen of this cypress, who were experiencing allergic rhinitis, conjunctivitis, or asthma, with the presence of specific IgE and positive skin prick tests. It shows a high degree of homology with Jun a 3 [33].

#### 4.2.5. Jun v 3

*Juniperus virginiana* is a source of TLP with a molecular weight of 10 kDa. Exposure occurs via inhalation [12].

#### 4.2.6. Can s 7

Cannabis also contains a TLP. In a group of 60 patients with sensitization confirmed by skin prick tests and the presence of cannabis-specific IgE, 27 patients had antibodies directed against TLP—Can s 7 [34]. In another study, out of 44 individuals with positive skin prick tests for *Cannabis sativa*, 8 showed the presence of antibodies to TLP [35].

### 4.3. Other Allergens

TLP has also been identified as a major allergen of *Manilkara zapota*. The identification was prompted by the evaluation of patients with oral allergy symptoms following fruit ingestion, positive skin test results, and the presence of specific IgE. An acidic TLP with a molecular weight of 26.9 kDa and a basic TLP with a molecular weight of 24.5 kDa were isolated [36].

In another case report, a group of 20 Finnish patients were evaluated. These patients were diagnosed with baker’s asthma, occupational allergic rhinitis, or both conditions and had positive skin prick tests or elevated wheat-flour-specific IgE levels. Extended diagnostics revealed the presence of antibodies to wheat TLP in nine of them [37].

There is also the possibility that a thaumatin-like protein with a molecular mass of 24 kDa has been identified as the cause of complaints such as occupational asthma or allergic rhinitis in patients who have been exposed to *Triplochiton scleroxylon* wood dust. In patients with rhinitis, 100% showed reactivity for TLP in basophil activation tests, and the ELISA test was positive in 92% of cases [38].

The tomato NP24 protein, which occurs in two isoforms, is also classified as a TLP. It is the subject of cross-reactivity studies involving TLP-group proteins [39].

TLP from lettuce was isolated through the analysis of a group of 42 patients with lettuce allergy. More than 50% of the tested sera reacted with TLP [40].

Kaki fruit, a novel allergen with a molecular weight of 16 kDa, was identified as a TLP protein. It was determined to be the cause of urticaria, angioedema, and rhinitis in a 34-year-old female patient who consumed kaki fruit [41].

A case of food-dependent anaphylaxis that was induced by the ingestion of oranges was also described, with TLP being identified as a possible cause of the reaction [42].

**Figure 1:**
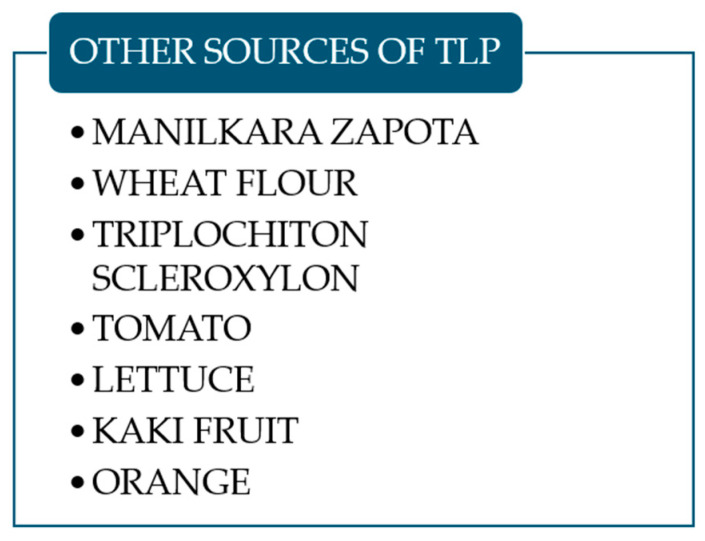
Other known sources of thaumatin-like proteins.

Other known sources of thaumatin-like proteins are listed in Figure 1.

## 5. Clinical Presentation

The clinical picture of allergy to TLP is not sufficiently detailed in the available medical literature. To date, researchers have not conducted systematic analyses that could bring us closer to a comprehensive understanding of the characteristics of this allergy and its various clinical manifestations. Currently, there are no unambiguous, well-defined symptoms that would form a coherent set, allowing us to distinguish the so-called “TLP syndrome”. The available data in the literature are primarily based on descriptions of clinical cases, which provide an incomplete representation of the issue.

The complaints experienced by patients who are allergic to TLPs range from local symptoms to severe anaphylactic reactions. Pollen food allergy syndrome (PFAS), whose main symptoms are swelling and itching of the lips, tongue, and throat, is one of the manifestations. Cross-reactions between cypress pollen TLPs and fruit TLPs can cause PFAS [43]. However, symptoms are not necessarily limited to the oral cavity, and anaphylaxis may also occur. Symptoms such as urticaria, angioedema, and gastrointestinal disturbances have been described [27].

TLP-induced anaphylaxis has been reported after the ingestion of kiwi, banana, and kaki [1,3,41]. A patient who was allergic to Act d 2 experienced swelling of the lips and face, pruritus of the skin, generalized urticaria, and significant weakness after kiwi consumption. The food intake was preceded by exercise. The diagnosis was confirmed by an oral food provocation challenge with a cofactor (physical activity). During the challenge, the patient developed an anaphylactic reaction in the form of urticaria, coughing, wheezing, and abdominal pain. The patient tolerated the kiwi fruit well without the cofactor [1].

Another patient experienced facial swelling and a feeling of tightness in her chest after eating a banana, while consuming grapes and pears caused tightness in her chest, itching in her throat and ears, vomiting, and abdominal pain. After eating tomato, apple, garlic, onion, carrot, chestnut, and lettuce, she experienced severe abdominal pain just a few minutes after consumption. This patient was found to have an elevated serum level of specific IgE for Act d 2, Mus a 4, chestnut TLP, and Platanus pollen TLP. In addition, skin prick tests were positive for Mus a 4 and Act d 2. Sensitization to other panallergens was also excluded [3].

A patient with confirmed sensitization to the TLP of sapodilla had oral allergy syndrome [36]. The kaki persimmon fruit was the cause of generalized urticaria, angioedema, rhinitis, abdominal pain, and shortness of breath in two patients. TLP-group protein and chitinase were identified as the cause of the allergy symptoms [41].

**Figure 2:**
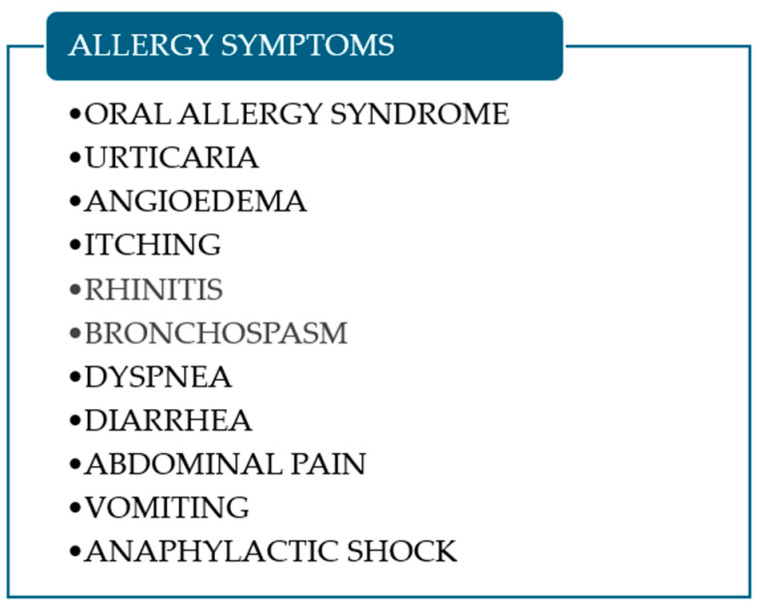
Symptoms of allergy to thaumatin-like protein.

Figure 2 summarizes the symptoms of allergy to thaumatin-like proteins.

## 6. Diagnostic Possibilities

The diagnosis of TLP sensitization is limited, and the availability of diagnostic tools is sparse.

The medical history is the first element of any diagnostic process. The symptoms that are caused by TLP allergy can be very diverse, ranging from oral allergy syndrome to full-blown anaphylaxis. The multiplicity of sources of TLP proteins, both described and presumably still unidentified, results in undiscovered cross-reactions. A detailed medical history helps identify the source of the allergen and provides insight into the progression of the reaction.

The next, but not less important, diagnostic step is skin prick testing, which in this case is only partially helpful. While skin prick tests can identify the source of the sensitizing allergen, they do not identify the specific allergen, which may be TLP proteins. Skin prick tests can be performed using ready-made allergenic extracts or fresh products. In one reported case, an acute anaphylactic reaction due to the ingestion of a banana was diagnosed using a skin prick test with TLP proteins—Mus a 4, Act d 2, and Pru p 2—isolated by laboratory methods [3]. However, this method is not commonly available in clinical practice. If made more widely accessible, it could help facilitate the diagnosis of all allergies.

After determining the source of the sensitizing allergen, assessing the presence of specific IgE still does not identify the exact allergen. Allergen-component-based diagnostics using the ImmunoCap ISAC multiparametric test can only detect the presence of specific antibodies to native Act d 2 (kiwi), while the ALEX2 Allergen Explorer test detects antibodies to both native Act d 2 (kiwi) and Mal d 2 (apple) [44].

Additionally, these tests assess Cry j, Cup s, Jun a, Can s, Cap a, Mus a, and Pru av; however, since these are allergen extracts, they still do not allow for the specification of the exact allergen. Unfortunately, more sensitive singleplex methods for the known TLPs are not available.

The methods described thus far only allow for the diagnosis of a few sources of TLPs. If the presence of sIgE for other allergens is excluded, it can only be assumed that the allergen that is responsible for the reaction is not available for the diagnosis of TLP.

**Figure 3:**
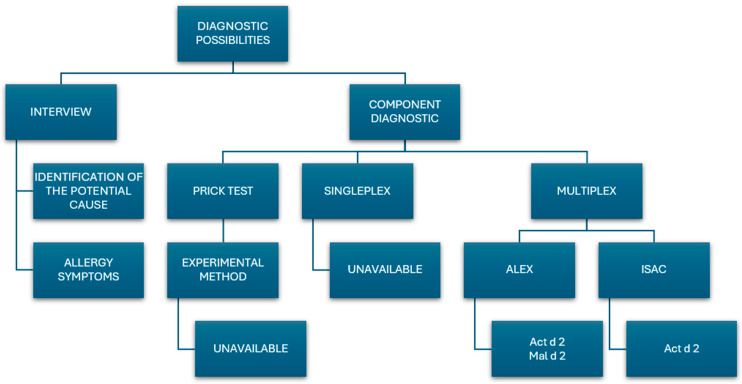
Diagnostic possibilities for allergy to thaumatin-like protein.

Figure 3 summarizes the possible diagnostic methods for allergy to thaumatin-like proteins.

## 7. Summary

Thaumatin-like proteins have been poorly studied. The descriptions that are available in the literature concern isolated cases, and the characterization of allergens and the course of sensitization appears incomplete. In this review article, the current state of knowledge on TLPs is summarized. Known allergens are described, and diagnostic options, although limited, are presented in detail. The clinical pattern of sensitization may differ, but in some cases, TLP allergy may manifest as anaphylaxis. For this reason, TLPs appear to be allergens of undervalued importance, and in cases of unclear etiology of allergic reactions following the consumption of fruits and vegetables, they should be considered.

Further studies are needed to assess the magnitude of the problem, the natural course of sensitization, and the prognosis for patients.

## Figures and Tables

**Table 1:** Currently described TLPs according to allergen.org [11,12].

Allergen	Source	Molecular Mass	Route of Exposure
Mal d 2	Apple	23 kDa	Oral
Pru p 2	Peach	25–28 kDa	Oral
Pru av 2	Sweet cherry	23 kDa	Oral
Cap a 1	Bell pepper	23 kDa	Oral
Act d 2	Green kiwi	24 kDa	Oral
Mus a 4	Banana	20 kDa	Oral
Ole e 13	Olive	23 kDa	Inhalation
Jun v 1	Eastern red cedar	10 kDa	Inhalation
Jun a 3	Mountain cedar	30 kDa	Inhalation
Cup s 3	Common cypress	34 kDa	Inhalation
Cry j 3	Japanese cedar	27 kDa	Inhalation
Can s 7	Indian hemp		Inhalation

## Data Availability

No new data were created or analyzed in this study. Data sharing is not applicable to this article.
